# Gender Differences in CDC Guideline Compliance for STIs in Emergency Departments

**DOI:** 10.5811/westjem.2016.12.32440

**Published:** 2017-03-08

**Authors:** Bryan G. Kane, Alexander W. D. Guillaume, Elizabeth M. Evans, Terrence E. Goyke, Jessica K. Eygnor, Lauren Semler, Stephen W. Dusza, Marna Rayl Greenberg

**Affiliations:** Lehigh Valley Hospital and Health Network/USF MCOM CC & I-78, Department of Emergency Medicine, Allentown, Pennsylvania

## Abstract

**Introduction:**

Sexually transmitted infections (STIs) are a common reason for emergency department (ED) visits. The objective of this study was to determine if there were gender differences in adherence to Centers for Disease Control and Prevention (CDC) STI diagnosis and treatment guidelines, as documented by emergency providers.

**Methods:**

We performed a retrospective chart review to identify patients treated for urethritis, cervicitis, and pelvic inflammatory disease (PID) in the EDs of three hospitals in a Pennsylvania network during a calendar year. Cases were reviewed to assess for compliance with CDC guidelines. We used descriptive statistics to assess the distributions of study variables by patient sex. In the analysis we used Student’s t-tests, chi-square tests, and logistic regression. Statistical significance was set at p ≤ 0.05.

**Results:**

We identified 286 patient records. Of these, we excluded 39 for the following reasons: incorrect disease coding; the patient was admitted and treated as an inpatient for his/her disease; or the patient left the ED after refusing care. Of the 247 participants, 159 (64.4%) were female. Females were significantly younger (26.6 years, SD=8.0) than males (31.2, SD=11.5%), (95% confidence interval [CI] [2.0– 7.0], p=0.0003). All of the males (n=88) in the cohort presented with urethritis; 25.8% of females presented with cervicitis, and 74.2% with PID. Physician compliance for the five CDC criteria ranged from 68.8% for patient history to 93.5% for patient diagnostic testing, including urine pregnancy and gonorrhea/chlamydia cultures. We observed significant differences by patient sex. Fifty-four percent of the charts had symptoms recorded for female patients that were consistent with CDC characteristics for diagnostic criteria compared to over 95% for males, OR=16.9; 95% CI [5.9–48.4], p<0.001. Similar results were observed for patient discharge instructions, with physicians completely documenting delivery of discharge instructions to 51.6% of females compared to 97.7% of complete documentation in males, OR=42.3; 95% CI [10.0–178.6] p<0.001). We observed no significant sex differences in physician documentation for physical exam or for therapeutic antibiotic treatment.

**Conclusion:**

This retrospective study found patient gender differences in how emergency providers complied with documenting with regard to the 2010 CDC guidelines for the diagnosis and treatment of urethritis, cervicitis, and PID. Specifically medical records of men were more likely to have complete documentation of symptoms recorded (95% CI 5.9–48.4) and to have discharge instruction documentation (95% CI 10.0–178.6) than records of women.

## INTRODUCTION

According to a recent report by the Centers for Disease Control and Prevention (CDC), the rate of infection of sexually transmitted diseases (STIs) is rising; as compared to 2013, the rates of infection of chlamydia and gonorrhea increased by 2.8% and 5.1%, respectively in 2014.^1^ This amounted to over 1.4 million cases of chlamydia and over 350,000 cases of gonorrhea reported in 2014.^1^ STIs are a common reason for emergency department (ED) visits. 2 In fact, up to 7% of patients seeking treatment for STIs do so in EDs.^3^ Furthermore, each year there are 171,000 adolescent ED visits for STIs.^4^ Women who develop a chlamydial or gonorrheal infection of the upper genital tract, also known as pelvic inflammatory disease (PID), are at increased risk for long-term sequelae including infertility and ectopic pregnancy.^2^ Men with untreated STIs can continue to transmit infections to others.^5^ Due to both the high incidence of ED visits for STIs and their potentially serious complications, ED providers must be adept at properly diagnosing and treating them.

The need to recognize and adjust for sex and gender differences is a growing topic in medical research. In fact, in 2015 the National Institutes of Health announced a new funding policy, stating that sex must be accounted for as a biological variable in research designs submitted after January 25, 2016.^6^ Despite this increasing focus on gender differences, a recent literature review found that only 18% of emergency medicine (EM)-related studies examined health outcome by gender, and that only 2% of studies included gender in the primary hypothesis.^7^ Additionally, this review determined that EM-based gender research lagged behind many other medical specialties including general medicine, cardiology, and oncology. Because EDs experience 136 million patient visits annually, emergency physicians (EP) have the opportunity to be a leading source of gender-based research that will optimize and individualize outcomes for patients in the acute care setting.^8^ In respect to terminology, while “sex” is the preferred term in basic science research, in this manuscript we have used the terms male/female or “gender” – referring to the socially constructed biological roles of an individual based on their XX or XY status, as it is more commonly used by clinicians.

We chose to assess for gender differences in physician compliance with the CDC’s 2010 guidelines for diagnosing and treating STIs. In particular, we evaluated EP-documented compliance across five domains – -history, physical exam, diagnostic testing, antibiotic treatment, and discharge instructions – to assess for differences by patient gender.

The objective of this study was to perform a chart review to determine if there were gender differences in adherence to CDC STI diagnosis and treatment guidelines as documented by emergency providers for the diagnoses of urethritis, cervicitis, and PID in the ED.

**Population Health Research Capsule**

What do we already know about this issue?

Sexually transmitted infections (STIs) are a common reason for emergency department (ED) visits.

What was the research question?

Are there gender differences in compliance with CDC STI diagnosis and treatment guidelines as documented by emergency providers?

What was the major finding of the study?

Physicians were more likely to document compliance with CDC guidelines for evaluation and treatment of STIs (95% CI 5.9–48.4) and to have discharge instruction documentation (95% CI 10.0–178.6) for men compared to women.

How does this improve population health?

Raising awareness may serve as a catalyst to improve patient care. Our findings should encourage clinicians to be more attentive to documentation.

## METHODS

Following network institutional review board (IRB) approval, we performed a retrospective chart review to identify all patients treated for urethritis, cervicitis, and PID in the EDs of three affiliated hospitals in Northeastern Pennsylvania from January 1, 2011, to December 31, 2011. These three sites have a combined ED census of greater than 185,000 visits per year (Table 1). Patients were identified by International Classification of Diseases (ICD-9) discharge diagnosis code. Included in the study were discharged patients over the age of 14 with the following ICD-9 codes: 098.00 through 098.39 for gonorrhea; 099.50 and 099.53 through 099.55 for chlamydia; 597.80 and 597.89 for urethritis; 614.90 for PID; and 616.0 for cervicitis. We reviewed the included cases as described using standard methods for chart reviews^9^ to assess for compliance with 2010 CDC guidelines that were used in the ED setting and determined to be essential for documentation across five domains: 1) history; 2) physical exam; 3) diagnostic testing including urine human chorionic gonadotropin (hCG), and gonorrhea and chlamydia cultures; 4) antibiotic treatment; and 5) discharge instructions. The study principle investigator trained abstractors (EM residents and attendings, coordinators and research associates) using explicit protocols of inclusion and exclusion before data collection was initiated and variables were precisely defined. A standardized abstraction form (IRB approved) was used to guide data collection. Regular meetings with the study team (including abstractors) to review project status occurred during the study period. Abstractors were aware of the protocol to detect compliance with CDC guidelines for STIs but were blinded to the study goal of determining gender differences. Each chart was reviewed again by a single research associate who completed a second abstraction form. Abstracted data from both reviews were assessed by one senior (attending physician) study investigator, and any minor discrepancies were resolved by a third chart review by the senior study investigator. Because the abstraction process was extensive (all charts reviewed twice, some three times), we did not conduct formal statistical analysis of interrater reliability.

Of note, this was a secondary analysis of a prior study that analyzed whether compliance with CDC guidelines improved when an electronic medical record (EMR) was implemented as compared to a study in which handwritten, non-templated charting was used. 10, 11

**Figure 1:**
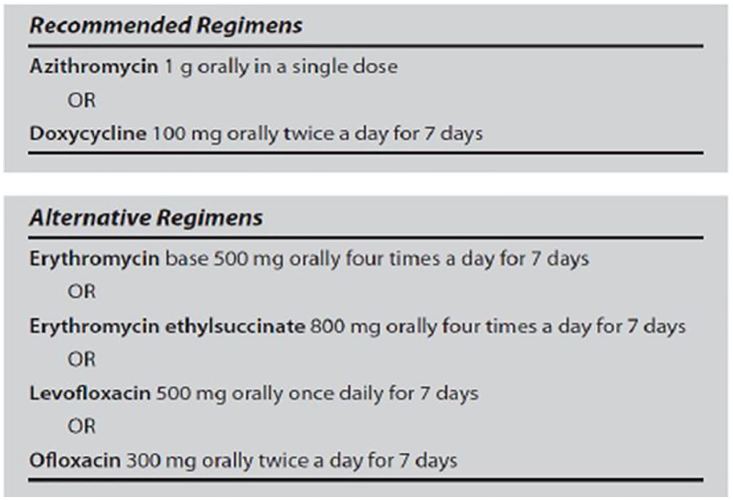
2010 urethritis and cervicitis guidelines^5^.

**Figure 2:**
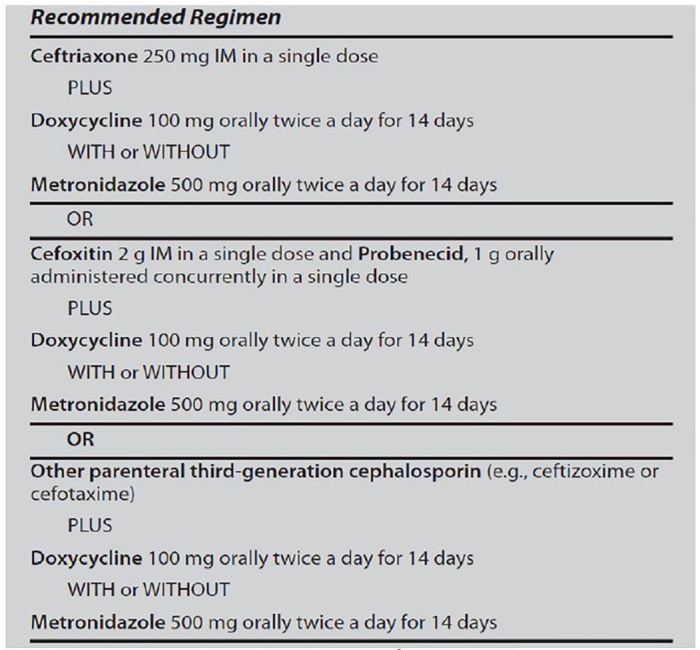
2010 CDC guidelines for treatment of pelvic inflammatory disease^5^. *CDC*, Centers for Disease Control and Prevention.

We assessed compliance with 2010 CDC standards across five domains for cases of urethritis, cervicitis, and PID.^5^ The outcomes evaluated included documentation of historical and diagnostic components, diagnostic testing, treatment provided, and follow-up instructions. Specifically, for urethritis, historical components included dysuria, urethral pruritus, or discharge. Physical exam findings were discharge of mucopurulent or purulent material. Diagnostic testing included obtaining cultures for gonorrhea and chlamydia. Antibiotic regimens were compared with CDC standards (Figure 1). Proper discharge instructions required safe-sex instructions. For cervicitis, diagnostic components included having a mucopurulent or friable cervix along with a history not suggestive of PID; patients must not have experienced a history of abnormal vaginal bleeding, dyspareunia, vaginal discharge or abdominal pain. Similarly, physical exam documentation required ruling out findings for PID, including a documented fever or experiencing any tenderness of the lower abdomen, adnexa or with cervical motion. Diagnostic testing included obtaining cultures as well as a pregnancy test. Antibiotic regimens and discharge instructions were the same as for urethritis. For PID, historical components and physical exam findings applied appropriately were as listed for cervicitis above. Diagnostic testing included cultures and a pregnancy test. Antibiotic regimens were compared with CDC standards (Figure 2). Discharge instructions included safe-sex instructions, follow-up, and return instructions.

We used descriptive statistics and graphical methods to assess the distributions of study variables by patient sex. Student’s t-tests and chi-square tests were used to evaluate the associations between study variables and patient sex. In addition, we used logistic regression to assess differences in physician documentation of the CDC 2010 guidelines by patient sex while controlling for patient age. Statistical significance was set at p ≤ 0.05. We performed all analyses with Stata v14.0, Stata Corporation, College Station, TX.

## RESULTS

We identified a total of 286 patient records. Of these, we excluded 39 due to incorrect disease coding (n=14), or because either the patient was admitted and treated as an inpatient for his/her disease (n=22) or left the ED after refusing care (n=3). The resultant sample size included 247 participants. Of the 247 participants, 159 (64.4%) were female. Females were significantly younger (26.6 years, SD=8.0) than males (31.2, SD=11.5%), (95% confidence interval [CI] [2.0–7.0], p=0.0003). By definition, all of the males (n=88) in the cohort presented with urethritis, whereas 25.8% of females presented with cervicitis, and 74.2% with PID.

The distribution of physician documentation compliance with CDC 2010 guidelines for treatment of STIs by patient sex are presented in Table 2. Physician compliance for the five CDC criteria ranged from 68.8% for patient history to 93.5% for patient diagnostic testing including urine *human chorionic gonadotropin (*hCG), gonorrhea, and chlamydia cultures. We observed significant differences by patient sex. Fifty-four percent of physician histories for female patients were compliant with the CDC guidelines, compared to over 95% for males, OR=16.9; 95% CI [5.9–48.4], p<0.001. Similar results were observed for patient discharge instructions, with physicians completely documenting delivery of discharge instructions to 51.6% of females compared to 97.7% of complete documentation in males, odds ratio (OR)=42.3; 95% CI [10.0–178.6], p<0.001. Documentation for diagnostic testing was marginally higher for female patients (96.8% versus 87.5%, p=0.011). We observed no significant sex differences in physician documentation for physical exam or for therapeutic antibiotic treatment.

## DISCUSSION

Our study identified significant patient gender differences in multiple aspects of emergency provider compliance with the 2010 CDC guidelines for the diagnosis and management of STIs. While it is reassuring that the therapeutic compliance category is not different between genders, the documented historical data and discharge instructions for males were significantly more compliant with CDC guidelines than for females. The finding that only 51.6% of females received CDC-compliant discharge instructions as compared to 97.7% of males is especially concerning. Because untreated chlamydia infections and cervicitis can progress to PID and incorrectly treated PID in women places them at risk for increased morbidity and mortality from conditions such as infertility or ectopic pregnancy,^2^ it is a compelling public health interest that women receive appropriate treatment, instructions for follow-up and reasons to return to the ED. Raising awareness and drawing attention to this problem through the results of this study optimally serves as a catalyst to potentiate improved patient care. Specifically, our findings should compel clinicians to be more attentive to documentation. For instance, we must be clear about the importance of documenting follow-up for these patients to prevent disease progression, incomplete treatment or untoward complications during pregnancy (ectopic). While necessary for pure clinical outcomes, documentation is also important from a medical legal perspective: absence of documentation is interpreted as absence of care delivered.

One could argue that compliance was lower in history and discharge documentation for PID as compared to urethritis because PID required more data points in these two domains. However, PID also required more data points for physical exam documentation, yet our study found no sex difference in this domain. Furthermore, cervicitis and PID both required more documentation for diagnostic testing than did urethritis, yet in this domain EPs were actually found to be more compliant for females than males (96.8% versus 87.5%). Therefore, these unaccounted-for gender differences in historical data and discharge instructions illustrate real yet unexplained trends that require further research before they can be fully understood and resolved.

Poor physician compliance with CDC guidelines for PID has been documented elsewhere in the literature. In 2011, Shih et al. examined over 1.6 million patients discharged from EDs nationwide with the diagnosis of PID and found that ED providers were only adherent with CDC antibiotic guidelines in 30.5% of cases.^12^ Although that study did not compare its outcomes to those of male patients, it does coincide with our findings that physicians require more familiarity with essential guidelines to ensure better patient care. In terms of gender differences in guideline compliance, a 2014 study by Manteuffel et al. analyzing the pharmacy claims for 29.5 million U.S. adults determined that women were consistently less likely to be prescribed guideline-based medications for diabetes and cardiovascular conditions.^13^ Most strikingly, they found that of the adults with coronary artery disease, only 59% of women were prescribed a cholesterol-lowering medication as compared to 71.5% of men.^13^ The unexplained gender differences found in our study echo those found in this much larger study, indicating a real disparity that might very well impact the clinical outcomes of those affected.

There is also a growing body of EM literature demonstrating gender differences in many common EM topics. Chen et al. found that women presenting to the ED with acute abdominal pain were 13–25% less likely than men to receive opioid analgesia.^14^ Additionally, they found that the women who did receive opioids waited on average 16 minutes longer than men to receive them.^14^ Furthermore, a study by Chang et al. determined that men presenting with acute coronary syndrome were more likely to undergo cardiac catheterizations (adjusted OR,1.72; 95% CI [1.40–2.11]) and stress tests (adjusted OR, 1.16; 95% CI [1.01–1.33]) than women.^15^ Finally, a recent study by Ryoo et al. that surveyed 27.9 million U.S. ED visits related to drug use determined that men were more likely than women to be referred to detoxification programs, even after controlling for patients who presented “seeking detox.”^16^ Although there is a growing recognition of gender differences in EM, very little else has been published about gender differences in STI management in the ED. Thus, our findings help highlight yet one more aspect of EM that requires further analysis to identify and alleviate gender differences. Interestingly, the historical cohort our study emulated also found that women were less likely to receive appropriate discharge instructions for cervicitis.^10^ This indicates that despite the use of a templated EMR, gender differences continue to exist in discharge instructions for patients treated for STIs.

Of note also is the difference in diagnostic testing compliance (more consistent in female patients than male). It is unclear why this difference exists, and this offers an area of opportunity for future study. There are multiple other directions to consider for future study. Most pressingly, there is a continued need to identify and correct gender differences in documentation (particularly discharge instructions) to prevent adverse medical outcomes. Additionally, evaluation of the CDC guidelines themselves for gender differences should be reviewed. At first blush, a persistent difference between recommendations regarding HIV/syphilis testing is present in both the 2010 and the newer 2015 CDC guidelines for urethritis and cervicitis (i.e., men have formal recommendation and women do not).^5^,^17^

It might also be worthy to assess how physician gender interacts with patient gender to affect outcomes. Some progress has been made in this area already. A prospective, multicenter study of 840 patients by Safdar et al. found that male EPs were more likely to prescribe opioids to males, and that female EPs were more likely to prescribe opioids to females.^18^ Furthermore, Napoli et al. found that male EPs were significantly less likely to stress-test female patients with chest pain (OR, 0.82; 95% CI [0.68–0.99]).^19^ With regard to gender differences in STI guideline adherence, future studies might discover interesting findings about how physicians’ own genders might impact their comfort in correctly performing exams, and level of comfort in discussing STIs with different patient genders.

## LIMITATIONS

One limitation of our study was that it was geographically restricted to three hospitals in Northeastern Pennsylvania. It is unclear how our study population compares to the overall population of individuals with STIs in the U.S. as a whole; thus, these results might not be generalizable. We also studied only care in the ED; it is unknown if patients presenting to the ED for these ICD-related complaints fundamentally are different from patients presenting to a primary care office or urgent care with similar symptoms. Additionally, we do not know what impact that having a female/male provider had on the findings in our study; we were unable to assess this because the majority of patients had multiple providers (residents, mid-level providers, and/or attending physicians). Also, this is a convenience sample and the impact of the sampling method on the results we report is unknown.

Furthermore, we did not study HIV/syphilis or trichomonas; the outcomes of physician documentation for these diseases are not known. Also, the impact of subtle (asymptomatic) differences in clinical presentations between men and women on physician documentation is not known. Furthermore, no effort was made to control for the difference in disease complexity between urethritis and PID. Additionally, there may have been some cases in which delivered care and follow-up instructions were done and not documented. Additionally, in the time frame between study initiation and completion, the CDC revised their recommendations for diagnosis and treatment of STIs.^17^ While there were few overall changes between the 2010 and 2015 guidelines, the impact on the study outcomes is not defined. Additionally, because our study was a retrospective chart review, it is subject to the limitations inherently found in these types of studies.

## CONCLUSION

This retrospective study found patient gender differences in how emergency providers complied with documenting with regard to the 2010 CDC guidelines for the diagnosis and treatment of urethritis, cervicitis, and PID. Specifically medical records of men were more likely to have complete documentation of symptoms recorded (95% CI 5.9–48.4) and to have discharge instruction documentation (95% CI 10.0–178.6) than records of women.

## Figures and Tables

**Table 1:** Hospital site comparisons in study of gender-based differences in following guidelines for STI treatment.

Site	Annual ED census	Character
1	100, 000	Level 1 trauma center
2	45,000	Suburban hospital
3	20,000	Inner city hospital

*STI*, sexually transmitted infections; *ED,* emergency department

**Table 2:** Distribution of compliance with CDC 2010 guidelines for treatment of sexually transmitted disease by patient sex.

Compliance criteria categories	Coding	Female	Male	Total	OR (95% CI)*	p-value
History						
	No (ref)	73 (45.9%)	4 (4.6%)	77 (31.2%)	--	--
	Yes	86 (54.1%)	84 (95.5%)	170 (68.8%)	16.9 (5.9–48.4)	<0.001
Physical exam						
	No (ref)	49 (30.8%)	20 (22.7%)	69 (27.9%)	--	--
	Yes	110 (69.2%)	68 (77.3%)	178 (72.1%)	1.5 (0.8–2.7)	0.222
Diagnostics						
	No (ref)	5 (3.2%)	11 (12.5%)	16 (6.5%)	--	--
	Yes	153 (96.8%)	77 (87.5%)	230 (93.5%)	0.2 (0.1– 0.7)	0.011
Therapeutic						
	No (ref)	12 (7.6%)	8 (9.1%)	20 (8.1%)	--	--
	Yes	147 (92.5%)	80 (90.9%)	227 (91.9%)	0.8 (0.3–2.0)	0.592
Discharge instructions						
	No (ref)	77 (48.4%)	2 (2.3%)	79 (32.0%)	--	--
	Yes	82 (51.6%)	86 (97.7%)	168 (68.0%)	42.3 (10.0–178.6)	<0.001

*CDC*, Centers for Disease Control and Prevention; *OR*, odds ratio.

* All odds ratio estimates were adjusted by participant age. In the models, females were coded as zero and males coded as 1. The odds ratios are to be interpreted as the odds of charting compliance for male relative to female patients.
